# The Association between Sleep Disturbances, Imaging Biomarkers and Cognitive Decline along the Trajectory of Alzheimer's Disease

**DOI:** 10.1002/alz70856_099181

**Published:** 2025-12-24

**Authors:** Reihaneh Ahmadi

**Affiliations:** ^1^ Institute of Neuroscience and Medicine, Jülich Research Center, Jülich, Jülich, Germany

## Abstract

**Background:**

Alzheimer's Disease (AD) is a prevalent and debilitating neurodegenerative condition that significantly impacts global health. Increasing evidence indicates that sleep disturbances, including insomnia and Sleep‐Disordered Breathing (SDB), may act as modifiable risk factors for AD. Despite extensive research into AD biomarkers, the complex interactions between sleep disturbances, neuroimaging biomarkers, and cognitive decline across the AD continuum remain insufficiently explored. This study aimed to investigate the relationships between self‐reported sleep disturbances, specifically insomnia and SDB, and imaging biomarkers associated with AD.

**Method:**

Data from 1510 participants in the Alzheimer's Disease Neuroimaging Initiative (ADNI) were analyzed. Imaging biomarkers, including tau and amyloid Positron Emission Tomography (PET) and structural Magnetic Resonance Imaging (MRI), were examined alongside self‐reported sleep disturbances and cognitive performance scores from the Alzheimer's Disease Assessment Scale ‐ Cognitive Subscale (ADAS‐Cog). Statistical analyses explored the effects of sleep disturbances on imaging biomarkers and cognitive outcomes while controlling for demographic and clinical factors.

**Result:**

Comparative analyses revealed that overall sleep disturbances and their interaction with AD diagnosis significantly impact tau deposition in key AD‐sensitive brain regions, including the amygdala, fusiform, inferior temporal and parietal cortices, and posterior cingulate cortex (corrected *p*‐value < 0.05). These regions are particularly vulnerable during the middle stages of AD. Distinct patterns emerged for insomnia and SDB, with SDB showing a more pronounced and earlier influence on both AD pathology and cognitive decline compared to insomnia. The findings suggest that SDB may be a more critical factor in accelerating AD progression, emphasizing the need for early intervention.

**Conclusion:**

This study demonstrates that sleep disturbances significantly contribute to tau burden and cognitive decline in AD, with SDB exhibiting an earlier and more pronounced impact than insomnia. These findings highlight the importance of targeting sleep disturbances to mitigate tau‐related pathology, protect cognitive function, and slow disease progression. Early diagnosis and targeted interventions for sleep disturbances could provide significant therapeutic benefits in managing AD and improving patient outcomes.

